# The Molecular Mechanisms of Neuroinflammation in Alzheimer’s Disease, the Consequence of Neural Cell Death

**DOI:** 10.3390/ijms241411757

**Published:** 2023-07-21

**Authors:** Su-Bin Choi, Sehee Kwon, Ji-Hye Kim, Na-Hyun Ahn, Joo-Hee Lee, Seung-Hoon Yang

**Affiliations:** Department of Biomedical Engineering, College of Life Science and Biotechnology, Dongguk University, Seoul 04620, Republic of Korea; dahee1546@dgu.ac.kr (S.-B.C.); shkwon@dgu.ac.kr (S.K.); kimjihye010216@dgu.ac.kr (J.-H.K.); skgus9506@dgu.ac.kr (N.-H.A.); 2018111727@dgu.ac.kr (J.-H.L.)

**Keywords:** Alzheimer’s disease, neural cell death, apoptosis, autophagy, neuroinflammation, necroptosis, NLRP3 inflammasome

## Abstract

Alzheimer’s disease (AD) is accompanied by neural cell loss and memory deficit. Neural cell death, occurring via apoptosis and autophagy, is widely observed in the AD brain in addition to neuroinflammation mediated by necroptosis and the NLRP3 inflammasome. Neurotoxicity induced by amyloid-beta (Aβ) and tau aggregates leads to excessive neural cell death and neuroinflammation in the AD brain. During AD progression, uncontrolled neural cell death results in the dysregulation of cellular activity and synaptic function. Apoptosis mediated by pro-apoptotic caspases, autophagy regulated by autophagy-related proteins, and necroptosis controlled by the RIPK/MLKL axis are representative of neural cell death occurred during AD. Necroptosis causes the release of cellular components, contributing to the pro-inflammatory environment in the AD brain. Inordinately high levels of neural cell death and pro-inflammatory events lead to the production of pro-inflammatory cytokines and feed-forward hyper neuroinflammation. Thus, neural cell death and neuroinflammation cause synaptic dysfunction and memory deficits in the AD brain. In this review, we briefly introduce the mechanisms of neural cell death and neuroinflammation observed in the AD brain. Combined with a typical strategy for targeting Aβ and tau, regulation of neural cell death and neuroinflammation may be effective for the amelioration of AD pathologies.

## 1. Introduction

The hallmarks of Alzheimer’s disease (AD) are the abnormal aggregation of amyloid-beta (Aβ) peptides and hyperphosphorylated tau proteins in the brain, resulting in the synaptic loss of neural cells and cognitive decline [1]. Aβs released from neural cells are aggregated into oligomers and fibrils, forming Aβ plaques. Hyperphosphorylated tau proteins formed by different kinases, including p38, c-Jun N-terminal kinase (JNK), microtubule-affinity regulating kinase (MARK), and protein kinase A (PKA), also aggregate to form abnormal structures of paired helical filaments and neurofibrillary tangles (NFTs) [2]. Aberrant Aβ and tau aggregates are resistant to proteolysis by several proteases, including calpain and the carboxyl terminus of Hsc70-interacting protein (CHIP), which leads to the accumulation of these highly neurotoxic aggregates in the brain. Once Aβ- and tau-mediated pathological changes begin to appear in the cortical or hippocampal regions of the AD brain, they spread to neighboring regions [3]. Several types of neural cell death, including apoptosis and autophagy, are often observed in the AD brain with neurotoxicity induced by Aβ and the tau aggregates [4].

Apoptosis is a type of programmed cell death that is involved in the development of the nervous system and modulates its homeostasis even after maturity [5]. It is morphologically characterized by cell shrinkage, membrane blebbing, chromatin condensation, and DNA fragmentation, forming apoptotic bodies with intracellular organelles. Upon recognition and engulfment by phagocytes, apoptotic neural cells do not leak their cellular components. Thus, impaired neural cells die with minimal harmful effects on adjacent cells and minimal induction of immune responses through apoptosis.

Autophagy is a lysosomal degradation process crucial for neural survival and is a precisely compartmentalized process [6]. It is a key pathway to clear abnormally aggregated proteins and organelles, ensuring protein homeostasis and neuroprotection. Neural cells possessing abnormal components such as damaged organelles and excessive protein aggregates form autophagic vacuoles called autophagosomes. Autophagosomes then fuse with lysosomes, separating normal and damaged components and resulting in their degradation and recycling for new biosynthesis. As the loss of neural cells is associated with the accumulation of abnormally aggregated proteins, the autophagic process is crucial for the function of neural cells.

Necroptosis, a regulated form of necrosis, is initiated by the activation of death receptors (DRs) such as the tumor necrosis factor (TNF) receptor superfamily [7]. Their morphologies are similarly characterized by the loss of cell membrane integrity and swelling of cells. Necroptotic cells secrete pro-inflammatory cytokines and leak their intracellular contents. The released cellular components circulate as inflammatory signals for the activation of the immune response of neighboring cells, which, in turn, induces the activation of neuroinflammation mainly in the microglia and astrocytes in the brain for host defense.

As neural cell death and neuroinflammation are often observed in the AD brain under uncontrolled conditions, neural cells consistently produce pro-inflammatory cytokines, resulting in feed-forward hyperinflammation [8]. Therefore, the regulation of neural cell death and neuroinflammation, as well as the clearance of Aβ and tau aggregates, are crucial targets in AD treatment. In this review, we briefly introduce neural cell death and neuroinflammation observed in AD development and discuss potential anti-cell death and anti-neuroinflammation strategies for the alleviation of AD pathology.

## 2. Functional Consequences of Neural Cell Death in AD

### 2.1. Pathological Signaling Mechanisms Induced by Apoptosis in the AD Brain

In various physiological processes, apoptosis is induced by pro-apoptotic factors such as B cell lymphoma-2 (BCL-2), BCL-2 associated X (BAX), p53, and pro-apoptotic caspases. The pro-apoptotic signaling pathways stimulated by cell stress lead to mitochondrial dysfunction and trigger caspase activation. These events lead to specific changes in cellular morphology, including plasma membrane blebbing, cell shrinkage, and nuclear fragmentation, in the brain [9].

**Figure 1:**
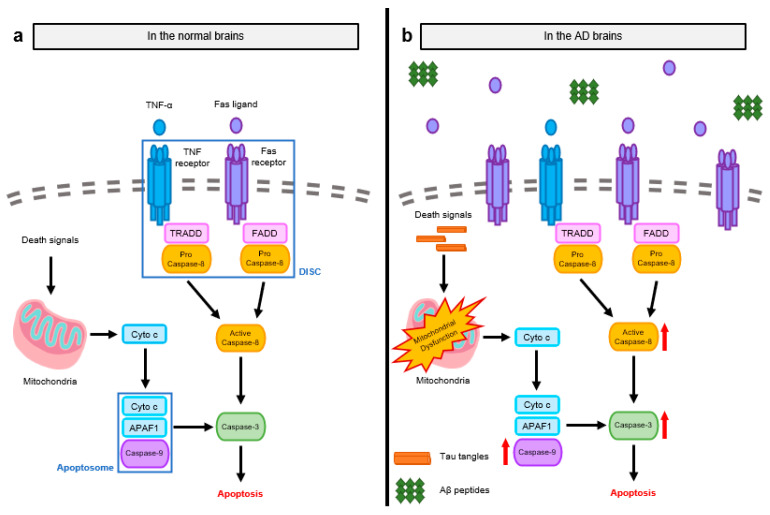
Molecular mechanisms of apoptosis observed in normal and AD brains. (**a**) Apoptosis is transduced by two main signaling pathways: intrinsic pathway mediated by mitochondria and extrinsic pathway mediated by DRs. After the initiation of apoptosis, signaling transduction is dependent on the activation of pro-apoptotic caspases. Caspase-9, an initial caspase in the intrinsic pathway, is activated by Cyto c and APAF1, assembling apoptosomes. Caspase-8, an initial caspase in the extrinsic pathway, is activated by the oligomerization of DRs and the subsequent recruitment of adaptor proteins, forming the DISC. Activated caspase-9 and -8 then trigger the activation of executioner caspase-3, leading to apoptosis in the neural cells. (**b**) In AD brains, as apoptosis is not appropriately regulated, both intrinsic and extrinsic pathways are elevated. Initial caspases are more activated by abnormal protein aggregates and increased Fas ligands. The activation of initial caspases induces the activation of caspase-3 with the large occurrence of apoptosis during AD.

Apoptosis is initiated by the activation of caspases via two main pathways: intrinsic pathway (mitochondrial pathway) and extrinsic pathway (death receptor-mediated pathway). The initiator caspase in the intrinsic pathway is caspase-9, which binds to apoptotic protease activating factor 1 (APAF1) and cytochrome c (Cyto c). Cyto c released from mitochondria into the cytosol induces conformational changes of APAF1 by binding to its adenosine triphosphate (ATP)/deoxyadenosine triphosphate (dATP)-binding oligomerization domain. These changes induce the exposure of the caspase recruitment domain (CARD) of APAF1 and consequent assembly with caspase-9, forming a functional complex called an apoptosome [10]. Activated caspase-9 triggers the activation of the executioner caspase-3, resulting in the apoptosis of astrocytes and neurons (Figure 1a) [11].

The extrinsic pathway of apoptosis is initiated by the accumulation of DRs, including Fas receptor, TNF receptor (TNFR), and TNF-related apoptosis-inducing ligand (TRAIL). After oligomerization of DRs by binding to death ligands such as TNF and Fas ligand, adaptor proteins, Fas-associated death domain protein (FADD), or TNFR1-associated death domain (TRADD) are recruited to the DRs. Then, monomeric procaspase-8 binds to these adaptor proteins, forming the signaling complex called the death initiating signaling complex (DISC). The activation of DISC triggers the cleavage and consequent activation of procaspase-8 followed by the activation of the downstream caspase family member, caspase-3 [12]. Activated caspase-3 then cleaves the inhibitor of caspase-activated DNase (ICAD), whose function is to block the activity of caspase-activated DNase (CAD). Free CAD released by the activation of caspase-3 degrades chromosomal DNA within the nuclei, causing neural cell death through chromatin condensation [5].

Unlike the normal brain, the AD brain generally shows neural cell death induced by abnormal Aβ and tau accumulation, leading to synaptic loss and further cognitive impairment. Neural cells undergo cell death with morphological features similar to apoptosis (e.g., cell shrinkage and DNA fragmentation [13,14]), altering the levels of apoptosis-associated proteins in the brains of AD patients [15]. The initial caspases involved in apoptosis, such as caspase-8 and caspase-9, are activated to a greater extent in the AD brain [16]. Caspase-8 is activated by stimulating Fas receptors, which are overexpressed in neurons in the AD brain [17], and caspase-9 is activated by mitochondrial dysfunction during AD development. The elevated activation of these initial caspases is followed by the activation of their downstream caspase family member, caspase-3. This series of caspase activation events indicates that neuronal apoptosis largely occurs during AD progression (Figure 1b).

To elucidate how Aβ and tau lead to neural apoptosis, several studies were performed using neural cell culture and AD brain tissues stimulated with Aβ and tau proteins. Aβ is known to cause apoptosis depending on its degree of aggregation [18]. Aβ peptides and fibrils cause apoptosis through the activation of caspase-8 and caspase-3 but not caspase-9, indicating that cell death is induced via the Fas signaling-induced extrinsic pathway [19,20]. Aβ fibrils also indirectly increase apoptosis by enhancing the expression of Fas ligands [21]. However, Aβ oligomers can also induce apoptosis via the intrinsic pathway by causing mitochondrial dysfunction [22]. As these oligomers have membrane permeability, they localize close to the mitochondria and damage the mitochondrial membrane to release Cyto c. Neurons treated with tau proteins also exhibit both intrinsic and extrinsic apoptotic signaling. Notably, phosphorylated tau initially activates caspase-9 in the pretangle neurons, suggesting that tau-induced cell death appears in the early stage of AD development [19]. In the case of the extrinsic neural cell death pathway induced by tau, Fas receptors activated by tau peptides and NFTs recruit FADD, which can activate caspase-8 and other kinases to additionally phosphorylate tau proteins and aggravate AD pathologies. Furthermore, treatment with Aβ and tau proteins upregulates pro-apoptotic BCL-2 family proteins, including BAX, BOK (BCL-2 related ovarian killer), and BAD (BCL-2 associated agonist of cell death), which also mediate mitochondrial dysfunction in the AD brain [23,24]. These pro-apoptotic proteins translocate from the cytosol to the mitochondria in the hippocampus and cortical neurons, inducing the leakage of Cyto c from the mitochondria to trigger the intrinsic apoptotic pathway via activation of caspase-3.

Apoptosis further affects AD pathologies through the catalytic activities of caspases. For example, activated caspase-3 can directly cleave amyloid precursor protein (APP), producing additional APP fragments with cytotoxicity [25]. These fragments accumulate in the cortical and hippocampal regions of the brain as AD progresses. Caspase-3 can also disrupt the cytoskeleton of neurons through cleavage of tau and fodrin (a membrane-associated cytoskeletal protein), leading to synaptic dysfunction of neurons and the alteration of neural cell morphology [26]. Furthermore, caspase-3 impairs AMPA (α-amino-3-hydroxy-5-methyl-4-isoxazolepropionic acid) receptors, which play a crucial role in synaptic plasticity and transmission. Caspase-3 cleaves the subunits of AMPA receptors to inhibit neuronal responsiveness to the neurotransmitter, glutamate. As a result, AD patients and AD transgenic mouse models show severe spatial learning and memory deficits due to neuronal loss and neurodegeneration [25,27].

### 2.2. Pathological Signaling Mechanisms Induced by Autophagic-Dependent Cell Death in the AD Brain

Macroautophagy (hereafter referred to as autophagy) was originally regarded as a cell survival mechanism for the degradation and recycling of cellular waste. However, autophagy also plays a pivotal role in neural cell death. Autophagy-dependent cell death (ADCD) is widely defined as a regulated cell death that depends on autophagic machinery in the absence of other forms of cell death such as apoptosis and necroptosis [6]. As the entire mechanism of ADCD signaling is not fully understood, many studies are underway to determine how autophagy leads to neural cell death.

**Figure 2:**
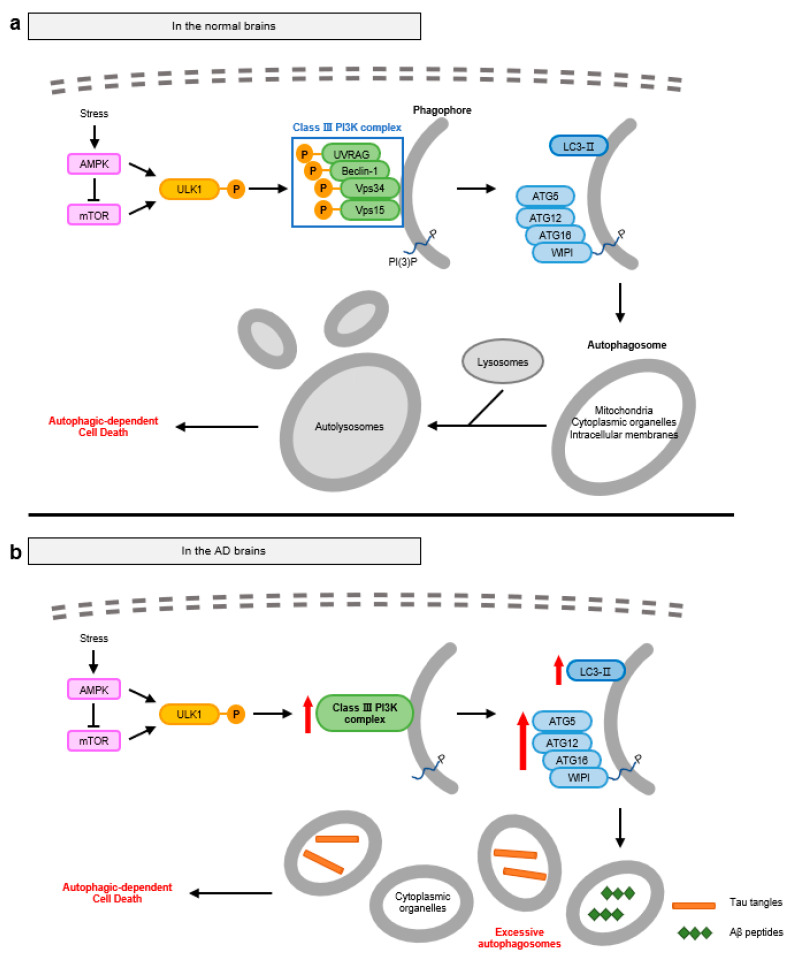
Molecular mechanisms of autophagy-dependent cell death observed in normal and AD brains. (**a**) Autophagy is initiated by the activation of ULK1 and the subsequent formation of class III PI3K complex, which generates phagophores. Class III PI3K complex then recruits ATG proteins to lead to the maturation of autophagosomes. Mature autophagosomes undergo fusion with lysosomes, becoming autolysosomes. Unlike the autophagy system for cell survival, neural cells that underwent ADCD excessively degrade cytoplasmic components such as mitochondria and endoplasmic reticulum, leading to ADCD in neural cells. (**b**) In AD brains, impaired neurons excessively generate autophagosomes in response to abnormal protein aggregates with increased levels of ATG and LC3-II proteins. However, as the clearance systems of autophagosomes are disrupted, enormous autophagosomes cannot fuse with lysosomes, continuously accumulated in the neurons, resulting in ADCD during AD.

Autophagy is naturally suppressed by adenosine monophosphate-activated protein kinase (AMPK), which activates mammalian target of rapamycin (mTOR) and inhibits Unc-51-like autophagy activating kinase 1 (ULK1) activity. In response to the intracellular stress or signals such as starvation and growth factor deprivation in the neural cells, AMPK directly activates ULK1 via phosphorylation or indirectly via the deactivation of mTOR [28,29]. Activated ULK1 triggers the formation of the class III phosphatidylinositol-3-kinase (PI3K) complex. The class III PI3K complex, which consists of vacuolar protein sorting 34 (Vps34), Vps15, Beclin-1, and UV radiation resistance associated gene protein (UVRAG), generates PI-3-phosphate (PI(3)P) and recruits several autophagy-related (ATG) proteins, resulting in the biogenesis of phagophores. Then, WD repeats domain phosphoinositide-interacting protein 2 (WIPI2), a PI(3)-binding protein, binds with ATG16, ATG5, and ATG12 with the production of LC3-II through the conjugation of LC3-I with phosphatidylethanolamine (PE), forming the autophagosomes. ATG proteins mediate the maturation and accumulation of autophagosomes in the peripheral sites of neural cells [30]. The mature autophagosomes fuse with lysosomes, becoming autolysosomes [31]. Because proximity between autophagosomes and lysosomes is important for fusion efficiency, diverse proteins involved in the mobility of autophagosomes such as various cytoskeletal proteins, Ras-associated binding (RAB) GTPase, and soluble NSF attachment protein receptor (SNARE) proteins are also involved in the fusion process [32]. However, the mechanism underlying the role of autophagy after the formation of the autolysosomes, leading to neural cell death is still elusive. Neural cells that undergo ADCD have autophagosomes, which are increased in number and size compared with the autophagosomes for cell survival. With the degradation of mitochondria, cytoplasmic organelles, and intracellular membranes, it is suggested that failure of appropriate cargo selection leads to cell death (Figure 2a) [33].

In the AD brain, excessive accumulation of autophagosomes is one of the features observed in damaged neurons with dystrophic neuritis [34]. Both mRNA levels of autophagy-related proteins, including ATG, and the LC3-II/LC3-I ratio are elevated, which is the hallmark of autophagy activation [35,36,37]. Furthermore, excessive accumulation of autophagosomes in the AD brain displays morphological characteristics distinct from those observed in the normal brain. These autophagosomes have single or double membrane-limited vesicles consisting of granular or amorphous dense contents [34]. These morphologies are similar to those detected in impaired neurons during failure of autophagosome clearance. These findings suggest that excessive formation of autophagosomes and failure of autophagosome clearance are important features of the AD brain (Figure 2b).

Aβ interferes with the normal autophagy system in two ways: by enhancement of autophagosome initiation and by interruption of their clearance. As cellular waste, Aβ peptides and oligomers promote autophagy, and Aβ-induced reactive oxygen species (ROS) accelerate the formation of the class III PI3K complex [35,38]. This increase in the levels of the class III PI3K complex results in excessive accumulation of autophagosomes. Additionally, the fusion of autophagosomes with lysosomes is important for the degradation of cellular cargoes and the clearance of autophagosomes in the normal autophagy process. However, Aβ oligomers accumulate near lysosomes and damage the lysosomal membrane due to their ability to infiltrate biological membranes [39]. Proteolytic enzymes belonging to the aspartic, cysteine, or serine proteinase families are leaked from the disrupted lysosomes, inducing the deacidification of their internal environments and eventually blocking lysosomal fusion with autophagosomes and the subsequent degradation of autophagosomes [40]. In addition to Aβ, co-localization of NFTs and autophagosomes has been observed in the AD brain. Tau-mediated axonal swelling arises in the early stages of AD, interrupting the transport of autophagosomes to the perinuclear sites. Consequently, neurons show increased formation and impaired clearance of autophagosomes, leading to degradation of cellular organelles and neural cell death. Moreover, phosphorylated tau activates AMPK receptors, triggering autophagy signaling [41].

Autophagy dysfunction caused by excessive accumulation of Aβ and tau induces less efficiency of Aβ and tau clearance, eventually leading to neuroinflammation. It is reported that deficiency of ATG5 or Beclin-1 gene in microglial cells activated NF-κB signaling with the production of mitochondrial reactive oxygen species (ROS) and proinflammatory cytokines [42]. Another study revealed that knockdown of Atg7 or LC3 induced the activation of NLRP3 inflammasome, which then leads to the release of proinflammatory cytokine IL-1β in the microglial treated with Aβ aggregates [43]. Moreover, microglia specific ATG7 conditional knockout mice or Beclin-1 deficiency in Alzheimer transgenic mice showed increased neuroinflammation and neuronal loss in response to Aβ administration [43,44], suggesting that autophagy has an important role in the regulation of neuroinflammation during AD progression.

## 3. Functional Consequences of Neuroinflammation in AD

### 3.1. Pathological Signaling Mechanisms Induced by Necroptosis-Mediated Neuroinflammation in the AD Brain

Necroptosis is another type of cell death induced by the activation of DRs, especially TNFR1 [7]. Necroptosis is mainly induced after traumatic brain injuries and is a major mediator of neural pathologies. The initial pathway of necroptosis is similar to that of apoptosis before the activation of caspase-8. However, when caspase-8 is deficient or inactivated and the RIPK3 level is sufficiently high, RIPK1 binds to RIPK3 through the RIP homotypic interaction motif (RHIM) domain in the hippocampal neurons, resulting in the oligomerization and phosphorylation of RIPK3 [45,46]. Phosphorylated RIPK3 mediates the recruitment of MLKL and forms necrosomes composed of RIPK1, RIPK3, and MLKL. MLKL is phosphorylated on its C-terminus pseudokinase domain by RIPK3. Subsequently, MLKL undergoes conformational changes, translocates to the cell membrane, and induces necroptosis by directly rupturing the plasma membrane and releasing cellular components, also called damage-associated molecular patterns (DAMPs) [47]. RIPK3-dependent necroptosis is also induced by double-stranded RNA (dsRNA), lipopolysaccharide (LPS), cytokines, or other types of cellular stress [48]. Toll-like receptor 4 (TLR4) and TLR3 recognize LPS and dsRNA, respectively, and activate necroptosis through the RHIM domain of Toll/Interleukin-1 receptor-domain-containing adapter-inducing interferon-β (TRIF) [49]. Both type I and II Interferon also mediate RIPK3-induced necroptosis by the induction of the RHIM-mediated interaction between ZBP1 (Z-nucleic acid binding protein 1) and RIPK3.

**Figure 3:**
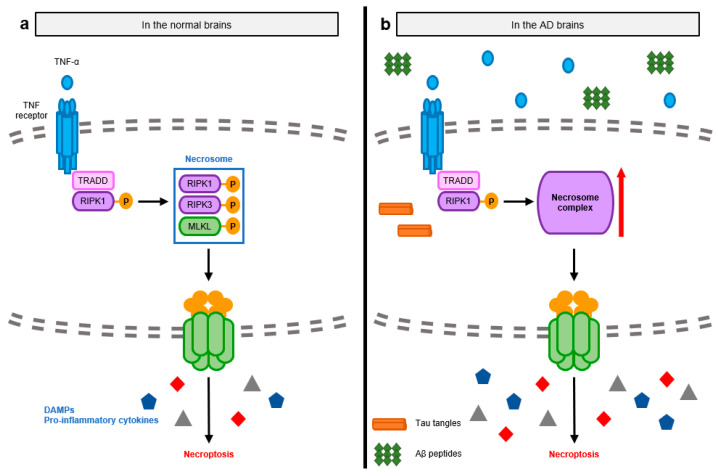
Molecular mechanisms of necroptosis-mediated neuroinflammation observed in normal and AD brains. (**a**) Necroptosis is induced by the activation of TNFR1. Oligomerized TNFR1 recruits and sequentially phosphorylated RIPK1 and RIPK3. Activated RIPK3 binds with MLKL, which is activated by the RIPK3-meidated phosphorylation, forming the necrosome complex. MLKL then migrates to the cell membrane and disrupts the membrane, causing the release of cellular organelles as DAMPs. Released DAMPs contribute to the amplification of pro-inflammatory responses in the brains. (**b**) In the early stages of AD, increased levels of TNF-α activate RIPK1-RIPK3-MLKL axis with extra formation of necrosomes, eliciting necroptosis in the brain. Subsequent enhancement of DAMPs is also elevated with the secretion of pro-inflammatory cytokines.

During necroptosis, dying cells simultaneously secrete bioactive pro-inflammatory cytokines such as Interleukin (IL)-1β and IL-33 in addition to the release of intracellular molecules to the extracellular environment [50,51]. Activated MLKL induces the efflux of intracellular potassium through membrane pores. Decreased potassium concentration leads to the activation of the inflammasome, promoting the secretion of mature pro-inflammatory cytokines [52]. The rupture of the membrane results in the release of DAMPs, which include the nucleus (e.g., DNA, histones, high motility group box 1 (HMGB1)), cytosol (e.g., uric acid and ATP), mitochondrial fragments and/or mitochondrial DNA, and plasma membrane. DAMPs are recognized by bystander microglia and astrocytes and act as circulating pro-inflammatory signals (Figure 3a) [53,54,55].

In the early stages of AD, the level of TNF-α is elevated in the brain, which initiates RIPK3-associated necroptosis by sequentially activating RIPK1, RIPK3, and MLKL [56,57]. This activation enhances their interactions, aiding in the formation of necrosomes in the hippocampal neurons [58,59]. During necroptosis, the release of DAMPs is likewise elevated, affecting blood brain barrier function and promoting the expression of pro-inflammatory cytokines in the AD brain [60]. These events lead to sterile neuroinflammation with a decrease in brain weight and cognitive ability (Figure 3b).

Aβ pathologies have been recently correlated with necroptosis, as neural cells with phosphorylated MLKL are spatially located around Aβ oligomers and plaques. Oligomeric Aβs trigger neuronal necroptosis by stimulating the microglia and astrocytes, which are tissue-resident immune cells in the central nervous system, for the clearance of DAMPs and abnormal protein aggregates [55,61,62]. Microglia and astrocytes recognize oligomeric Aβs and secrete TNF-α to activate necroptosis in neurons [55,63,64].

Vigorous necroptosis in the AD brain further elicits and aggravates neuroinflammation. In addition to the released DAMPs from necroptotic neurons triggering neuroinflammation, the RIPK1-RIPK3-MLKL axis is crucial for necroptosis-induced cytokine production in a cell-autonomous manner [51]. Notably, canonical nuclear factor-κB (NF-κB) signaling is elevated during necroptosis through the necroptosis-specific nuclear entry of p65, a component of NF-κB, and degradation of inhibitor of NF-κB α (IκBα), promoting the secretion of pro-inflammatory cytokines during necroptosis. Furthermore, these necroptosis-mediated neuroinflammation events exaggerate neuroinflammation by impairing the phagocytic activity of microglia. In the altered inflammatory environments of the AD brain, RIPK1 mediates a change to induce dysfunction in microglia, which are then called disease (or damage)-associated microglia (DAM) [65,66]. The kinase activity of RIPK1 disrupts phagocytosis through the transcriptional upregulation of Cst7, which encodes cystatin F blocking endosomal/lysosomal activation. DAMs are consequently defective in their degradation of abnormal protein aggregates and other DAMPs released during necroptosis, which further aggravates hyperinflammation in the AD brain.

### 3.2. Pathological Signaling Mechanisms Induced by Inflammasome-Mediated Neuroinflammation in the AD Brain

The inflammasome is an intracellular multiprotein complex that responds to pathogen-associated molecular patterns (PAMPs). The inflammasome mediates neuroinflammation via two steps—a priming step for the synthesis of the inactive forms of pro-inflammatory cytokines and the subsequent activation step for the maturation of these inactive forms [67].

**Figure 4:**
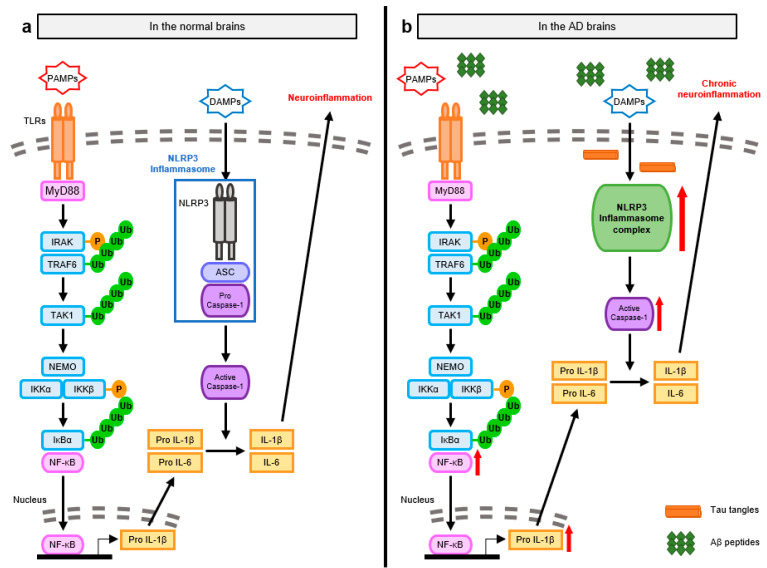
Molecular mechanisms of NLRP3 inflammasome-mediated neuroinflammation observed in normal and AD brains. (**a**) NLRP3 inflammasomes mediate neuroinflammation via two steps-a priming step and the subsequent activation step. In the priming step, microglia and astrocytes recognize PAMPs and transduce the TLR4-mediated MyD88-dependent NF-κB signaling. MyD88 further activates IRAK, TRAF6, and TAK1. Stimulated TAK1 then phosphorylates IKKβ and induces the ubiquitin-dependent degradation of IKKα, leading to the activation of NF-κB and the expression of inactive proinflammatory cytokines such as IL-1β and IL-18. These inactive proinflammatory cytokines are matured in the NLRP3 inflammasome platform. NLRP3 of microglia and astrocytes recognizes DAMPs, further recruiting ASC and pro-caspase-1 with the formation of NLRP3 inflammasomes. Caspase-1 activated in the NLRP3 inflammasome cleaves mature the inactive forms of the proinflammatory cytokines into bioactive form via their cleavage activity, resulting in neuroinflammation. (**b**) In AD brains, as the spreading pro-inflammatory cytokines and abnormal protein aggregates are recognized by microglia and astrocytes, both NF-κB and NLRP3 inflammasome are elevated. The activation of NLRP3 inflammasome contributes to the production of active pro-inflammatory cytokines with the aggravation of chronic neuroinflammation.

In the priming step, microglia and astrocytes recognize PAMPs, such as microbial molecules, through TLRs [55,68]. In particular, the TLR4-mediated myeloid differentiation factor 88 (MyD88)-dependent NF-κB pathway is crucial for the innate immune response in neural cells [69]. TLR4 recruits the downstream signaling molecule MyD88 via interaction with its Toll-IL-1 receptor (TIR) domain. MyD88 further associates with IL-1 receptor-associated kinase (IRAK) family members through their death domains. When IRAK family proteins are sequentially phosphorylated, IRAK dissociates from MyD88 and activates TNF receptor-associated factor 6 (TRAF6), an E3 ubiquitin ligase. Activated TRAF6 induces self-ubiquitination and the ubiquitination of NF-κB essential modifier (NEMO) [70]. TRAF6 also attaches ubiquitin chains to ubiquitin-dependent kinase transforming growth factor-β-activated kinase 1 (TAK1), which has two subunits, tubulin antisense-binding protein 1 and 2 (TAB1 and TAB2). Stimulated TAK1 phosphorylates IκB kinase β (IKKβ), which forms the IKK complex along with NEMO and IKKα. The IKK complex leads to the phosphorylation and ubiquitin-dependent degradation of IκBα and the activation of NF-κB, a key transcription factor involved in the expression of pro-inflammatory cytokines such as IL-1β and IL-18. These cytokines are produced in their biologically inactive forms in neural cells and mature via inflammasome platforms. After the priming step, the microglia and astrocytes recognize DAMPs from necrotic cells or the decrease in potassium concentration via NLRP3. Activated NLRP3 then recruits apoptosis-associated speck-like protein containing a CARD (ASC) through the pyrin domain and procaspase-1 through the CARD, forming the inflammasome [55,71]. When the death fold domains of procaspase-1 become self-associated, procaspase-1 undergoes self-cleavage and is processed to form mature caspase-1. Activated caspase-1 further cleaves the inactive forms of the pro-inflammatory cytokines into mature, bioactive forms, eliciting neuroinflammation in the brain (Figure 4a).

The NLRP3 inflammasome is considerably activated in the AD brain, contributing to the observed AD pathologies [72]. The trigger of NLRP3 inflammasome activation is significant for IL-1β maturation, aggravating chronic inflammatory responses. TLR4 of microglia and astrocytes is expressed at higher levels, forming more abnormal protein aggregates in AD transgenic mice compared to wild-type mice [8,55]. The enhancement of NF-κB signaling and its translocation are also observed in the neurons close to these abnormal protein aggregates [73]. The expression of inflammasome components, including NLRP3, ASC, and caspase-1, is also remarkably increased with the production of pro-inflammatory cytokines, IL-1β and IL-18 [74]. This indicates that AD-associated neuroinflammation is a sterile inflammation that activates a negative feedback loop for chronic neuroinflammation (Figure 4b).

During AD progression, Aβ can induce inflammasome-mediated neuroinflammation by stimulating both TLR4 and NLRP3. Microglia are progressively located close to Aβ deposits and are persistently stimulated by them. Abnormal Aβ aggregates and Aβ peptides are recognized by TLR4 and transduce the MyD88-dependent NF-κB signaling cascade. Aβ deposits further directly activate NLRP3 or indirectly activate it by producing other DAMPs [75]. For example, once Aβ deposits are phagocytosed and internalized into microglial lysosomes, Aβ-containing lysosomes then become swollen and undergo structural modifications, disrupting Aβ degradation. They lose their lysosomal integrity and release lysosomal enzymes, including cathepsin B, which function as DAMPs. Aβ deposits and the released DAMPs are recognized by intracellular NLRP3, forming the NLRP3 inflammasome strongly associated with Aβ-induced neuroinflammation. Tau tangles drive neuroinflammation mediated by NF-κB and NLRP3 inflammasome in microglia [76]. As the microglia engulf oligomeric tau and secrete them in exosomes after processing, tau tangles can spread from one brain region to another. This may also promote neuroinflammation and the seeding and spreading of tau proteins in the AD transgenic mouse model [77].

NLRP3 inflammasome signaling further advances other pathologies in AD. Activated NLRP3 recruits ASC in the form of assembled, helical fibrils. Extracellular ASC specks progressively bind with Aβ deposits in the AD transgenic mouse model [78]. The pyrin domain is the most important domain in ASC that is responsible for ASC assembly into helical fibrils. A mutation in the pyrin domain inhibits the effects of ASC specks on Aβ aggregation, suggesting that the assembly of ASC might be important for the interaction between ASC specks and Aβ. The formation of β-sheet-rich Aβ oligomers and fibrils is increased with higher levels of the extracellular helical form of ASC, indicating that ASC specks mediate the cross-seeding of Aβ in the early stages of Aβ aggregation and deposition. Additionally, the NLRP3 inflammasome mediates various disruptions in the AD brain by promoting the production of pro-inflammatory cytokines. IL-1β further induces NF-κB signaling in the microglia and triggers p38 mitogen-activated protein kinase (MAPK) signaling with the phosphorylation of tau proteins in neurons in the early stages of AD [76]. Moreover, IL-1β contributes to the disruption of synaptic plasticity in the AD transgenic mouse model. IL-18 increases the levels of APP and beta-site APP cleaving enzyme 1 (BACE1), aggravating the progression of AD via the acceleration of Aβ processing [72]. These cytokines create a feedback loop to further activate neuroinflammation for AD progression.

## 4. Discussion

Here, we have reviewed the molecular mechanisms of neural cell death and neuroinflammation observed in the AD brain: (a) apoptosis, (b) autophagy, (c) necroptosis-mediated neuroinflammation, and (d) NLRP3 inflammasome-mediated neuroinflammation. Aβ plaques and NFTs, major neuropathological hallmarks of AD, accumulate in the AD brain, as their clearance via autophagy and phagocytosis by microglia is disrupted. Neurotoxicity triggered by Aβ plaques and NFTs then elicits neural cell death and neuroinflammation, which are not appropriately regulated during AD. Additionally, since the brain does not regenerate new tissues during AD progression, irreversible neural loss and sustained neuroinflammation result in a loss of synaptic function and further memory deficit.

**Figure 5:**
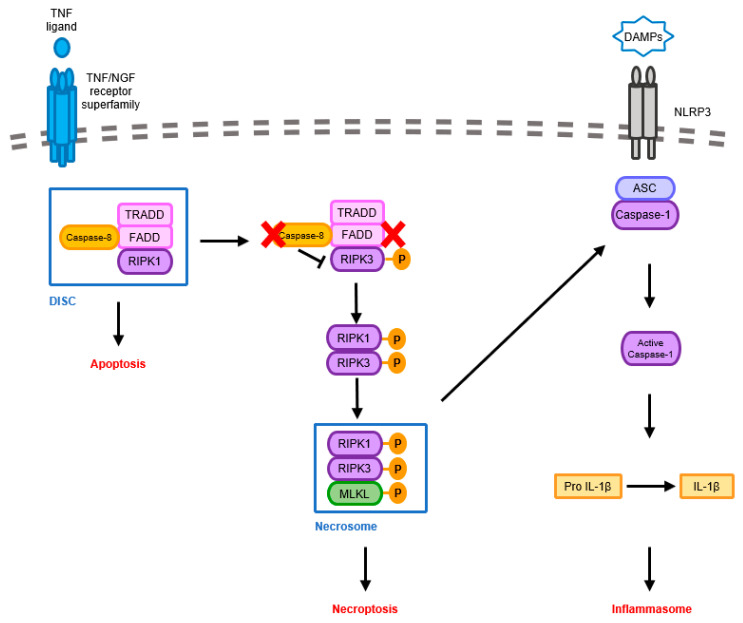
Molecular mechanisms of shared proximal signaling pathways by apoptosis, necroptosis, and NLRP3 inflammasome. The activation of TNF/NGF receptor superfamily recruits adaptor proteins and caspase-8 to form the DISC. This then triggers the transduction of apoptosis. However, when caspase-8 is inhibited by certain physiological conditions, indicated as a red X in the figure, this frees RIP kinases (RIPK1 and RIPK3) from restriction, and necrosome is formed to initiate necroptosis. The induction of necrosome is associated with NLRP3 inflammasome activated by DAMPs, leading to the production of IL-1β and inflammation.

Neuroinflammatory responses, including inflammasome activation, share proximal signaling pathways with the induction of neural cell death, such as apoptosis and necroptosis. For example, caspase-8 has been broadly known as an initiator caspase of the apoptotic signaling pathway, which is triggered by the TNF/NGF receptor superfamily. Upon stimulation, FADD, TRADD, and caspase-8 are recruited to form a signaling complex called DISC (Death Inducing Signaling Complex), eventually leading to apoptosis. When caspase-8 is inhibited by certain physiological conditions, RIP kinases (RIPK1 and RIPK3) form the necrosome to initiate another cell death pathway known as necroptosis. [79,80]. Many studies also suggested that caspase-8 negatively regulates the RIPK1/RIPK3-mediated activation of NLRP3 inflammasome [81,82]. (Figure 5) Additionally, increased evidence suggested that other types of neural cell death, such as pyroptosis, ferroptosis, and PANoptosis were observed in AD brains, suggesting certain types of cell death alone may not be associated with AD pathogenesis [80,83,84]. Thus, understanding the correlation between several types of neural cell death and neuroinflammation will provide a potential therapeutic strategy to overcome AD.

The molecular signaling mechanisms between neural cell death and neuroinflammation have been tightly orchestrated in the microglia and astrocytes of brains [55]. The activation of neuroinflammation caused by neural cell death is tightly regulated by several kinds of inhibitory proteins, such as cellular inhibitor of apoptosis protein (cIAP), inhibitor of caspase-activated DNase (ICAD), IκB kinase (IKK), and c-myc, etc. [85]. However, protein aggregates, including Aβ and tau in AD, can induce neural cell death and neuroinflammation like DAMPs [80]. Therefore, the regulation of excessive neuroinflammation should be considered in the development of AD therapeutics to maintain the balance of host immune systems.

To date, many drugs have been used to treat AD and restore memory deficit. For example, donepezil, an acetylcholinesterase inhibitor, is commonly used, and aducanumab, an Aβ-directed antibody, was approved by FDA under accelerated approval based on the decline of Aβ plaques in AD patients treated with aducanumab (Clinical trial ID. NCT02477800). However, as these agents only delay the symptoms rather than a complete recovery of brain functions, further preclinical studies must be carried out to identify strategies to regulate uncontrolled neural cell death and neuroinflammation. Thus, controlling neural cell death and neuroinflammation is also important for AD treatment in addition to the clearance of abnormal protein aggregates.

## Data Availability

Not applicable.
